# Molecular Cytogenetic Identification of the Wheat–*Dasypyrum villosum* T3DL·3V#3S Translocation Line with Resistance against Stripe Rust

**DOI:** 10.3390/plants11101329

**Published:** 2022-05-18

**Authors:** Jie Zhang, Shuyao Tang, Tao Lang, Ying Wang, Hai Long, Guangbing Deng, Qian Chen, Yuanlin Guo, Pu Xuan, Jun Xiao, Yun Jiang

**Affiliations:** 1 Institute of Biotechnology and Nuclear Technology Research, Sichuan Academy of Agricultural Sciences, Chengdu 610061, China; christine219@163.com (J.Z.); tangshuyao0609@sina.com (S.T.); langtao123xxx@126.com (T.L.); wangyinggzy@aliyun.com (Y.W.); chenliu83087@163.com (Q.C.); yuanlin_guo@163.com (Y.G.); xiaojun1919@aliyun.com (J.X.); 2 Key Laboratory of Wheat Biology and Genetic Improvement on Southwestern China (Ministry of Agriculture), Chengdu 610066, China; 3 Chengdu Institute of Biology, Chinese Academy of Sciences, Chengdu 610041, China; hailong@cib.ac.cn (H.L.); denggb@cib.ac.cn (G.D.); 4 Institute of Agro-Products Processing Science and Technology, Sichuan Academy of Agricultural Sciences, Chengdu 610066, China; xuanpu1964@163.com

**Keywords:** *D. villosum*, translocation line, stripe rust, molecular marker, ND-FISH

## Abstract

The annual species *Dasypyrum villosum* possesses several potentially valuable genes for the improvement of common wheat. Previously, we identified a new stripe rust-resistant line, the Chinese Spring (CS)–*D. villosum* 3V#3 (3D) substitution line (named CD-3), and mapped its potential rust resistance gene (designated as *YrCD-3*) on the 3V#3 chromosome originating from *D. villosum*. The objective of the present study was to further narrow down the *YrCD-3* locus to a physical region and develop wheat-3V#3 introgression lines with strong stripe rust resistance. By treating CD-3 seeds with ^60^Co γ-irradiation, two CS-3V#3 translocation lines, T3V#3S.3DL and T3DS.3V#3L (termed 22-12 and 24-20, respectively), were identified from the M_4_ generation through a combination of non-denaturing fluorescence in situ hybridization (ND-FISH) and functional molecular markers. Stripe rust resistance tests showed that the line 22-12 exhibited strong stripe rust resistance similarly to CD-3, whereas 24-20 was susceptible to stripe rust similarly to CS, indicating that *YrCD-3* is located on the short arm of 3V#3. The line 22-12 can potentially be used for further wheat improvement. Additionally, to trace 3V#3 in the wheat genetic background, we produced 30 3V#3-specific sequence tag (EST) markers, among which, 11 markers could identify 3V#3S. These markers could be valuable in fine-mapping *YrCD-3*.

## 1. Introduction

Bread wheat (*Triticum aestivum* L.) is a staple food crop whose global production has been challenged by many fungal diseases including stripe rust [1]. Stripe rust, caused by *Puccinia striiformis Westend. f.* sp. *tritici* Erikss. (*Pst*), was previously considered endemic in areas with low temperatures [2]; however, recently, its incidence has been reported in regions with warmer climate during the wheat-growing season [3,4,5]. Genetic resistance is the most effective measure to manage this disease [6]. To date, 83 stripe rust resistance genes (*Yr1* to *Yr83*) have been identified [6], and resistance to many of these genes’ products has been acquired by the pathogen. For example, *Yr9*, a rye-derived stripe rust resistance gene, which was widely used in Chinese wheat breeding programs in the 1960s, became ineffective due to the emergence and spread of the pathogen race CYR29, which was first documented in 1987 [7]. Afterward, during the 2008–2009 crop season, *Yr24/Yr26* resistance was acquired by a new race, V26 [7,8], which is also virulent to wheat lines harboring*Yr10* [7,9]. The wild relatives of common wheat carry valuable resistance genes for wheat diseases [10,11,12], for example, the rust resistance genes *Yr9/Sr31/Lr26* and *Yr83*, originated from *Secale cereale*; of these, *Yr9/Sr31/Lr26* is prevalent in commercial wheat cultivars in China [9,13,14].

*Dasypyrum villosum* (L.) Candargy (syn. *Haynaldia villosa* (L.) Schur, 2n = 2x = 14, VV) is an annual wild relative of common wheat and it is highly resistant to wheat diseases such as powdery mildew, eyespot, and stripe rust [15,16,17]. Although more than 300 *D. villosum* accessions have been collected [18], only 5 accessions (designated as V#1–V#5) have been used to produce wheat–*D. villosum* alien lines [19,20,21,22,23,24]. Among these lines, the *D. villosum*–common wheat 6V#2S.6AL translocation line has been widely applied in wheat breeding [25] for its well-known durable powdery mildew resistance gene, *Pm21*, located on 6V#2S of *D. villosum* [26]. Recently, *Pm21* has been cloned [27,28]. Studies have reported stripe rust resistance in some *D. villosum* populations [17]. However, limited progress has been made in the exploration of stripe rust resistance genes in *D. villosum* [29]. Previously, we characterized a novel CS-*D. villosum* 3V#3 (3D) substitution line, named CD-3, which exhibited strong stripe rust resistance [30]. The stripe rust resistance gene originating from *D. villosum*, namely, *YrCD-3*, was found to be located on the 3V#3 chromosome [30]. This indicated that CD-3 may be a valuable source of resistance genes to stripe rust in wheat, and its stripe rust-resistant gene (named *Yr CD-3*) should be exploited further.

Numerous molecular markers have been developed to monitor *D. villosum* chromatin in the wheat background [31,32], map favorable characteristics on chromosomal regions [12], and detect resistance genes located on the chromosomes originating from *D. villosum* [33,34]. However, only a few molecular markers are available for the utilization of *D. villosum* in wheat breeding. Transcriptome information based on next-generation sequencing (NGS) is a high-throughput and low-cost method that is widely used to develop molecular markers for simple sequence repeats (SSR) mining [35] and single-nucleotide polymorphism discovery [36]. Studies have reported the development of some molecular primers specific to *D. villosum*#4 chromosomes 1V to 7V based on RNA sequencing (RNA-seq) information [37].

Gamma irradiation is considered an effective method for inducing mutations [38], and this method has been widely used for transferring alien chromatin to the wheat genetic background [39], for wheat breeding [40], and for creating wheat–alien introgression lines to map and clone target genes [41]. In the present study, we treated CD-3 seeds with ^60^Co γ-irradiation and precisely identified 3V#3.3D translocation lines from the M_4_ generation; then, *YrCD-3* was mapped on the short arm of the 3V#3 chromosome. Finally, new EST markers were developed in the present study to monitor the 3V#3 chromosome arms in the wheat genetic background. 

## 2. Results

### 2.1. Generation and Screening for Wheat–D. villosum 3V#3.3D Translocation Stocks

A total of 315 seeds of CD-3 were irradiated by ^60^Co γ-rays in the present study. Fifteen seeds from each line (CD-3^200Gy^ and CD-3^ck^) were randomly selected and analyzed through conventional Feulgen staining for chromosomal behavior assessment. The results of the cytological analysis indicated large-scale structural aberrations of chromosomes and abnormal mitotic chromosomal behavior, for example, chromosome segments and chromatin bridges were observed in the line CD-3^200Gy^, whereas 42 chromosomes and no chromosomal fragment were observed in the line CD-3^ck^ (Figure S1A–D). The remaining 300 seeds from each line were sown in field plots to analyze their survival rate. The survival rates of CD-3^200Gy^ and CD-3^ck^ were found to be 75% and 98%, respectively, and the survival rate of CD-3^ck^ was significantly higher than that of CD-3^200Gy^ (*t*-test, *p* = 0.0011) (Figure S1E). These results indicated that mutants of this batch of CD-3 seeds were effectively induced by ^60^Co-γ ray.

Then, 1000 M_1_ plants were randomly selected and screened by V-genome-specific marker, DV1 [31], analyses, and the M_1_ plants carrying V-genome-specific bands were used for FISH analyses. In total, 563 M_1_ plants were successfully identified using FISH. We observed three types of chromosomal structural changes, involving 3V#3 chromosome (3V#3.wheat translocation (type 1), 3V#3.3V#3 translocation (type 2), and 3V#3 deletion (type 3)) in 79 plants (Figure S2A,B). The average inducing frequency was 14.03%. However, most of the aberration lines were heterozygous (i.e., they contained either one aberrant chromosome and one intact 3V#3 or two types of aberrant chromosomes). Most of the structural aberrations in the M1 plants could be transmitted to their progenies. More than 100 plants with only one translocation or deletion segregated in the M_2_–M_3_ progenies. From the self-fertilized progenies (M_4_) with the aforementioned aberrations, based on FISH and V-genome-specific marker analyses, two homozygous translocation lines for unique wheat-3V#3 translocations were isolated.

### 2.2. Identification of T. aestivum–D. villosum Structural Aberrations by ND-FISH

**Figure 1:**
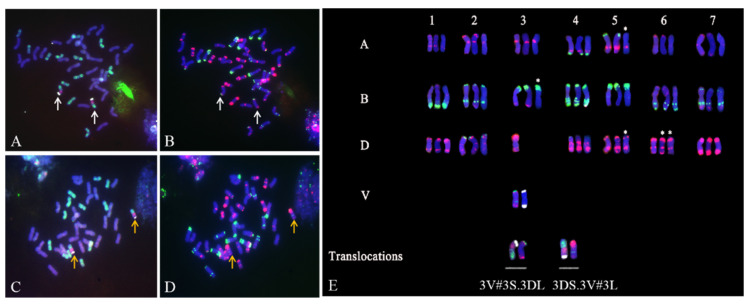
FISH patterns of 22-12 and 24-20. (**A**,**C**), Chromosomes of 22-12 (**A**) and 24-20 (**C**) stained with DAPI (blue), Oligo-Ku (red), Oligo-PHV62-1(white) and Oligo-D (green). (**B**,**D**), Chromosomes of 22-12 (**B**) and 24-20 (**D**) stained with DAPI (blue), Oligo-pTa535 (red) and Oligo-pSc119.2 (green). (**E**), FISH karyotype of individual chromosomes in CS (left), 22-12 (middle) and 24-20 (right); The A, B, D and V in (**E**) indicated A subgenome, B subgenome, D subgenome and 3V#3 chromosome, respectively; the wheat and 3V#3 chromosomes were stained with DAPI (blue), Oligo-pTa535 (red), Oligo-pSc119.2 (green) and Oligo-pHv62-1(white); the translocations (left) were stained with DAPI (blue), Oligo-Ku (red), Oligo-pHv62-1(white) and Oligo-D (green), and the translocations (right) were stained with DAPI (blue), Oligo-pTa535 (red), Oligo-pSc119.2 (green). The white arrows show 3V#3S.3DL, and the yellow arrows indicate 3DS.3V#3L. The asterisks indicate the chromosomes showing different signal patterns with respect to CS.

ND-FISH analysis using the probes Oligo-pHv62-1, Oligo-Ku, and Oligo-D showed that the lines 22-12 and 24-20 contained 42 chromosomes including a pair of 3V#3.D translocation chromosomes (Figure 1A,C). Subsequently, a second round of ND-FISH using the probes Oligo-pSc119.2 and Oligo-pTa535 was conducted on the same mitotic spread chromosomes of 22-12 and 24-20, as well as on the control CD-3 and CS. The results showed all wheat and 3V#3 chromosomes could be accurately distinguished using the probes Oligo-pSc119.2 and Oligo-pTa535 (Figure 1B,D). By comparing the standard wheat karyotype obtained using the probes Oligo-pSc119.2 and Oligo-pTa535 [42] and based on the results of the first round of FISH analysis, we inferred that the wheat–*D. villosum* translocations in 22-12 and 24-20 were 3V#3S.3DL and 3DS.3V#3L, respectively. 

Additionally, we observed that the signal patterns of some chromosomes in 22-12 and 24-20 differed from those in CS. For example, 5A in 24-20 exhibited a weak signal of Oligo-pSc119.2 in the terminal region of the short arm, not observed in CS. Additionally, 3B in 24-20 exhibited no Oligo-pTa535 signal at the end of the long arm, whereas 3B in CS carried an unmistakable Oligo-pTa535 signal at the end of the long arm. Similarly, 5D in 24-20 exhibited no Oligo-pTa535 signal at the end of the long arm, differently from CS. Notably, 6D in 24-20 harbored a barely visible Oligo-pTa535 signal at the end of the short arm, whereas 6D in CS exhibited an apparent terminal oligo-pTa535 signal on the short arm, and 6D in 22-12 carried a stronger oligo-pTa535 signal on the short arm compared to CS (Figure 1E).

### 2.3. Molecular Marker Analysis

**Figure 2:**
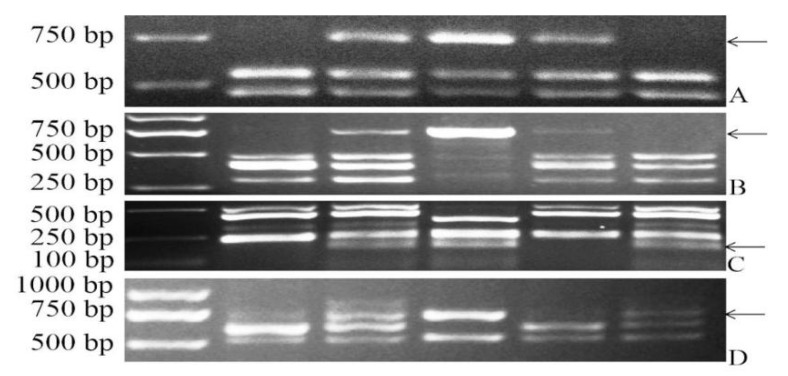
PCR products of PLUG markers in CS, CD-3, *D. villosum*, 22-12 and 24-20. (**A**–**D**) PCR amplification generated by the markers TNAC1248, TNAC1326, TNAC1267 and TNAC1359 respectively; M, Trans2k plus DNA markers (TransGen Biotech, Beijing, China); 1, common wheat CS “Chinese Spring”; 2, CD-3; 3, *D. villosum*; 4, 22-12; 5, 24-20. The arrows indicate *D. villosum*-specific bands.

The lines 22-12 and 24-20, their parent line CD-3, the positive control *D. villosum*, and the negative control CS were analyzed by employing 18 PLUG markers specific for wheat homoeologous group 3 chromosomes. Among the 18 PLUG primer pairs, two primer pairs (TNAC1241 and TNAC1326) generated *D. villosum*-specific bands in *D. villosum*, CD-3, and 22-12, whereas the other two primers (TNAC1267 and TNAC1359) amplified the *D. villosum* fragment from *D. villosum*, CD-3 and 24-20 (Figure 2). Ishikawa et al. [43] reported that TNAC1248 and TNAC1326 were located on the short arm, whereas TNAC1267 and TNAC1359 were located on the long arm (Table 1). Combined with the FISH analysis, we confirmed the line 22-12 as T3DL.3V#3S RobT and the line 24-20 as T3DS.3V#3L RobT.

### 2.4. Stripe Rust Response

**Figure 3:**
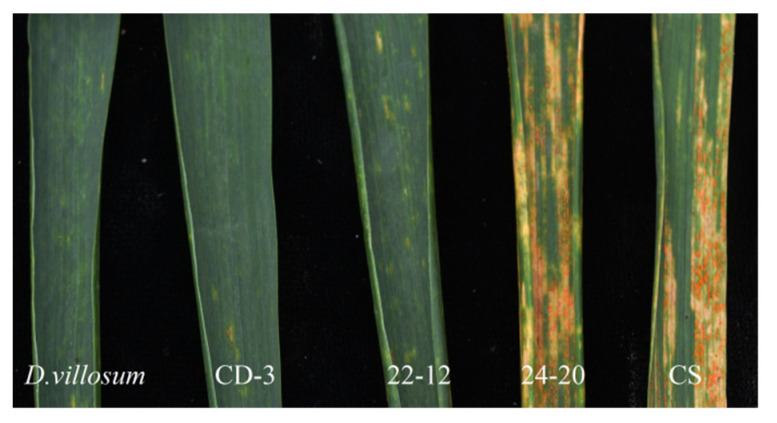
Phenotypic response to stripe rust of *D. villosum*, CD-3, 22-12, 24-20 and CS.

To identify the IT of the lines 22-12 and 24-20, the adult plant resistance (APR) test of stripe rust was conducted on all tested materials, including two CS–*D. villosum* 3V#3.3D translocation lines, CD-3, *D. villosum* accession PI 491576, and CS. The line 22-12 was found to be near immune to stripe rust (IT 0;), similarly to its parent line, CD-3, and the positive control *D. villosum*, whereas the line 24-20 exhibited moderate susceptibility (IT 3), similarly to the negative control CS (Figure 3). The stripe rust resistance gene YrCD-3, originated from *D. villosum* and therefore was mapped to the short arm of the 3V#3 chromosome.

### 2.5. Development and Verification of 3V#3-Specific EST Markers

**Figure 4:**
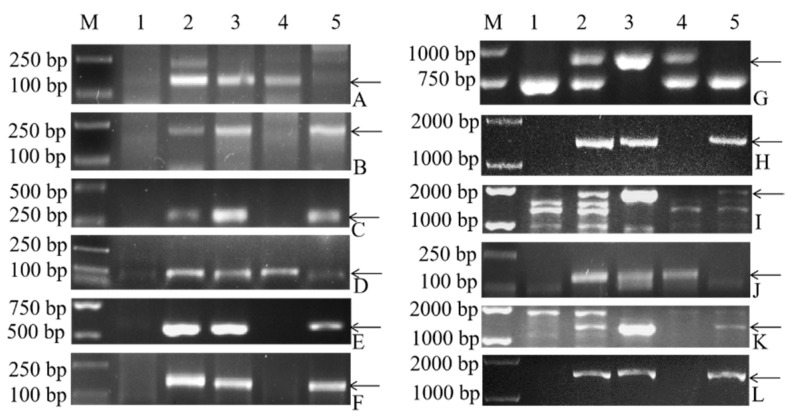
PCR patterns obtained with the primers 3V#3-1–3V#3-12 for wheat–*D. villosum* derivatives and their parents. M is Trans2K plus DNA marker, 1–5 are CS, CD-3, *D. villosum* accession PI 491576, 22-12 and 24-20, respectively. (**A**–**L**) Markers 3V#3-1–3V#3-12. The arrows showe *D. villosum*-specific bands.

By comparing the CD-3 sequences with *D. villosum* accession PI 491576 sequences acquired by RNA-seq, followed by the removal of the CS transcripts by reference genome matching, a subset of 3901 unigene sequences was obtained and used as putative 3V#3 chromosome-specific sequences. Based on the candidate sequences, we obtained 12 primer pairs, namely, 3V#3-1–3V#3-12, which could amplify stable, clear, and 3V-specific bands in CD-3 and *D. villosum*. The genomic DNA from CS, CD-3, *D. villosum* accession PI 491576, and the lines 22-12 and 24-20 was used to localize the new markers. Of the 12 primer pairs, 4 were found to be specific for 3V#3S (3V#3-1, 3V#3-4, 3V#3-7 and 3V#3-10), and the remaining 8 were found to be specific for 3V#3L (Figure 4, Table 2).

**Figure 5:**
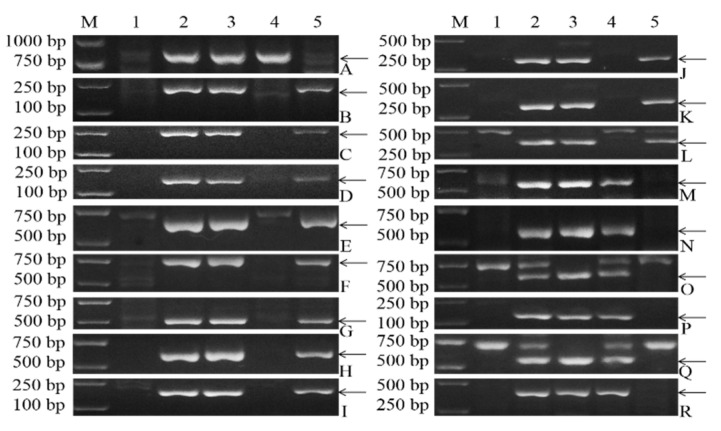
PCR patterns of 18 primers 3V#4 for wheat-*D. villosum* translocations and their parents. M indicates Trans2K plus DNA marker, 1–5 are CS, CD-3, *D. villosum* accession PI 491576, 22-12 and 24-20, respectively. (**A**–**R**) Markers 3V#4-1, 3V#4-2, 3V#4-3, 3V#4-5, 3V#4-7, 3V#4-11, 3V#4-12, 3V#4-14, 3V#4-16, 3V#4-17, 3V#4-18, 3V#4-19, 3V#4-20, 3V#4-21, 3V#4-22, 3V#4-24, 3V#4-26, 3V#4-27. The arrows show *D. villosum*-specific bands.

To produce high-density molecular markers to target the short arm and long arm of *D. villosum* chromosome 3V#3, PCR was performed on CS, CD-3 (3V#3), *D. villosum* accession PI 491576, and the lines 22-12 and 24-20 by using the EST primers specific to 3V#4 [37], which helped determine the effectiveness of these markers and localize them on 3V#3. Among the 27 3V#4-specific EST markers, 18 primer pairs could produce stable, clear and 3V#3-specific fragments in CS, CD-3 and *D. villosum* accession PI 491576. Eleven of 18 EST markers were mapped on the long arm of 3V#3, whereas the remaining 7 markers were mapped on the short arm of 3V#3 (Figure 5). Additionally, some products’ sizes in *D. villosum*#3 were not consistent with those in *D. villosum*#4, such as the sizes of the products amplified by 3V#4-1, 3V#4-3, 3V#4-7, 3V#4-20, 3V#4-21, and 3V#4-22. 

The results indicated that a total of 30 EST markers specific to chromosome 3V#3 could be used to characterize the 3V#3 chromosome’s arms. Among these markers, 11 markers could target 3V#3S. The markers are all specific and stable and thus valuable in detecting and localizing the novel stripe rust resistance gene *YrCD-3* on the *D. villosum* chromosome 3V#3S in wheat.

## 3. Discussion

*D. villosum* confers resistance to several wheat diseases such as powdery mildew, stripe rust, leaf rust, stem rust, eyespot, and take-all, as well as to wheat spindle streak mosaic virus (WSSM) [44,45]. Thus, it has been extensively used as a vital genetic resource for wheat breeding. Since the last century, several *D. villosum*–common wheat alien lines, such as amphidiploids [19], additional lines [20], substitution lines [21,46] and translocation lines [47,48,49], have been developed, and several resistance genes, such as *Pm62* [18], *Pm21* [50]*, Sr52* [51], have been explored and mapped on individual V-genome chromosomes by using wheat–*D. villosum* alien lines. In 2000, Yildirim et al. [17] tested the stripe rust reactions of *D. villosum* accessions, and the results showed that, of the 115 *D. villosum* accessions tested, 41 (35.65%) were resistant to stripe rust, indicating that *D. villosum* provides a vast pool of genes for stripe rust resistance. However, there only a few stripe rust resistance genes have been explored in *D. villosum* [52].

Gamma irradiation (by ^60^Co-γ) is an effective means to transfer many beneficial traits from the genomes of alien species to those of wheat [30,41]. In the current study, the seeds of CD-3 were treated with ^60^Co γ-rays to develop wheat–3V#3 translocation lines carrying strong stripe rust resistance conferred by *YrCD-3*. Chromosome fragmentation, chromatin bridges, and a substantial decrease in the survival rate were observed in the M_0_ generation, which was consistent with the findings of previous studies [38,53,54]. The results indicated that mutants of this batch of CD-3 seeds were effectively induced by ^60^Co-γ ray. Thus, the subsequent generations could be used as candidate populations for further screening the 3V#3.wheat translocation lines. In addition, several 3V#3 structural aberration lines were obtained from the irradiation progenies, and these chromosomal structural aberration lines could help for the further physical mapping of *YrCD-3* on the 3V#3 chromosomal region. 

Combined analysis based on FISH, V-genome-specific markers helped in identifying two new wheat–*D. villosum* translocation lines, namely, 22-12 and 24-20, from the M_4_ generation. FISH analysis showed that the line 22-12 was T3DL.3V#3S RobT, and the line 24-20 was T3DS.3V#3L RobT. Moreover, the presence of 3V#3S and 3V#3L in 22-12 and 24-20, respectively, was also confirmed by the subsequent PLUG marker analysis. The *Pst* resistance test at the adult stage indicated that 22-12 is highly resistant to stripe rust similar to CD-3 and *D. villosum*, whereas 24-20 is susceptible to stripe rust similar to CS. Thus, *YrCD-3*, derived from *D. villosum*, was mapped on the short arm of the 3V#3 chromosome, indicating that *YrCD-3* can be expressed in the genetic background of 22-12 (T3DL.3V#3S RobT). Furthermore, the novel T3DL.3V#3S RobT, 22-12, with a high degree of resistance to stripe rust, could be a valuable germplasm for wheat improvement.

ND-FISH has been widely used for identifying chromosomal structural variations of wheat and its relatives [38,55]. Some probes that can replace conventional genomic in situ hybridization (GISH) were developed to distinguish alien genomes in the wheat genetic background, including Oligo-B and Oligo-D for targeting the genomes B and D of wheat [56], Oligo-1162 for targeting the R genome of rye [55], Oligo-Ku for identifying the V-genome of *D. villosum* [57], and Oligo-B11 and Oligo-pThp3.93 for monitoring the E genome of *Thinopyrum* [58]. The present study used the probes Oligo-D and Oligo-Ku along with Oligo-pSc119.2, Oligo-pTa535, and the V-genome-specific probe Oligo-Hv62-1 to characterize the chromosomal structural variants in the lines 22-12 and 24-20. Oligo-D and Oligo-Ku could accurately identify the chromosome breakage–reunion occurring between V-genome and D-genome chromosomes. The probe Oligo-Hv62-1 could easily highlight 3V#3 chromosomes with strong signals, whereas the probes Oligo-pSc119.2 and Oligo-pTa535 could discriminate every individual chromosome and detect chromosomal structural changes. Chromosomal structural changes have been commonly observed in the progeny of the plants subjected to ^60^Co-γ-irradiation [38,53]. In the present study, slight FISH signal pattern changes, in addition to the translocation chromosomes, were observed in the lines 22-12 and 24-20 compared with the CS chromosomal karyotype. Large-scale mutants, such as deletion mutants and dicentromerics, which are commonly observed in the early generation (M_0_) [38,53], were not observed in the lines 22-12 and 24-20 (M_4_), indicating that substantial structural changes in somatic chromosomes are likely to be recovered to some extent. 

RNA-seq is a potential technique for identifying novel molecular markers in some plant species, especially those with limited existing genomic information. Markers based on transcription data have been developed to target the chromatin of wild wheat relatives transferred into the wheat genetic background. For example, *Thinopyrum intermedium* genome-specific EST-SSR markers [59], *Agropyron cristatum* chromosome 6P-specific EST markers [60], *Aegilops longissima* chromosome arm-specific PCR markers [61], rye-specific PCR markers [62], and *D. villosum*#4 chromosome 1V#4-7V#4-specific PCR markers [37,63] have been developed based on transcriptome data of wheat relatives. In the present study, 12 3V#3-specific EST markers were developed based on the transcriptome data of CD-3 and *D. villosum*. Of these, four markers were mapped to 3V#3S, whereas eight makers were mapped to 3V#3L. We also compared the locations of these markers on the wheat groups 1–7 and found that the locations of nine markers specific to 3V#3 chromosomes were consistent with their chromosomal locations in wheat, whereas two markers including 3V#3-2 and 3V#3-4 displayed different chromosomal locations in wheat. The present result showed that the high homologous sequence of marker 3V#3-2 mapped on 3V#3S was located on 1DS, whereas that of 3V#3-4 mapped on 3V#3S was located on chromosomes 1AL in wheat, which revealed that the collinearity of the developed markers on the chromosomal regions in *D. villosum*#3 and wheat was interrupted, and these genomic divergences were likely to drive the establishment of new species. A similar phenomenon was observed in a previous study [36]. Additionally, 27 3V#4-specific EST markers reported by Li et al. [37] were selected to test their effectiveness on the 3V#3 chromosome. Finally, only 18 primer pairs could produce stable, precise, and 3V#3-specific bands. Among them, 7 markers were mapped to 3V#3S, whereas 11 were mapped to 3V#3L. Additionally, five product sizes in *D. villosum*#3 were not consistent with those in *D. villosum*#4, indicating that genomic divergence occurred between *D. villosum*#3 and *D. villosum*#4 during the evolutionary process, which may be due to differences in the adaptation of the two *Dasypyrum* accessions to the environment. Therefore, the 30 PCR markers identified could potentially be used for tracing 3V#3 arms in the wheat genetic background, and the 11 3V#3S-specific markers could help for the further physical mapping of *YrCD-3* on the 3V#3 chromosomal region. 

## 4. Materials and Methods

### 4.1. Plant Materials

*D. villosum* accession PI 491576 (genome VV, 2n = 2x = 14) was obtained from the National Genetic Resources Program, United States Department of Agriculture. CS-*D. villosum* 3V#3 (3D) substitution line, namely, CD-3, is an F_6_ progeny derived from hybridization between CS and CS–*D. villosum* 3V#3 addition line, as described by Zhang et al. [30]. CD-3 and the common wheat CS were maintained by our laboratory. In total, 315 seeds of CD-3 were subjected to ^60^Co-γ irradiation, and 15 seeds from the mutagenized generation (M_0_) were selected randomly and used for evaluating the effectiveness of the irradiation treatment. The remaining 300 seeds were planted in the field. The subsequent generations (M_2_–M_4_) were used as candidate populations for screening and identifying the 3V#3.wheat translocation lines. The lines 22-12 and 24-20 were selected from the self-pollinated progenies (M_4_). A mixture of the cultivars Mingxian169 and SY95-71 was used as spreaders in the stripe rust test and was maintained by our laboratory.

### 4.2. ^60^Co γ-Irradiation Treatment of CD-3 Seeds

In total, 315 CD-3 seeds were treated by ^60^Co γ-irradiation at a dosage of 200 Gy, at a dosage rate of 1.0 Gy/m. The irradiation treatment was performed at the Institute of Biotechnology and Nuclear Technology Research, Sichuan Academy of Agricultural Sciences, China. 

### 4.3. Mitotic Analysis and Survival Rate Investigation of the M_0_ Generation 

Fifteen M_0_ generation seeds (named CD-3^200Gy^) were randomly selected for mitotic analysis, and 15 nonirradiated seeds of CD-3 were used as the control (named CD-3^CK^). The root tip preparation and mitotic studies were conducted using the methods described by Blanco et al. [64]. Cytological observations were performed using the Leica DM2500 microscope (Leica, Shanghai, China). 

The survival rate of the M_0_ generation was investigated in Pixian city, Sichuan, China, in 2017. A total of 600 seeds (300 seeds for each line, CD-3^200Gy^ and CD-3^ck^) were sown following an experimental block design with sixty 3 m rows (10 seeds per row). Three replicates (100 seeds from each line and a total of 200 seeds per replicate) were sown in the experimental block (3 m × 20 m). 

### 4.4. Statistics Analysis 

Statistical analyses were performed using GraphPad Prism version 8.01 (GraphPad Software, San Diego, CA, USA). Student’s *t*-test was used to estimate the statistical significance of the comparison, with a *p*-value of 0.05 as the threshold. 

### 4.5. FISH Analysis

To synchronize cell division, the seeds of CD-3, 22-12, and 24-20 were placed on wet filter paper at 23 °C for 24 h and then transferred to 4 °C for 24 h, followed by their transfer to 22 °C for 24 h. Root tips (approximately 2 cm long) were collected and then treated with nitrous oxide for 2 h and fixed with 90% acetic acid for 8–10 min. Afterward, the root tips were washed twice with ddH2O and stored in 70% ethanol at −20 °C. Chromosome preparation was performed according to the procedures described by Kato et al. [65]. The oligonucleotides Oligo-pSc119.2, Oligo-pTa-535 [42], V-genome-specific probe (Oligo-pHv62-1) [66], V-genome-specific probe (Oligo-Ku) [57], and D genome-specific probe (Oligo-D) [56] were used to identify individual chromosomes. These probes were synthesized by Invitrogen (Shanghai, China). ND-FISH analysis was performed as described by Fu et al. [55]. All images were captured using a Leica DM2500 microscope (Leica, Shanghai, China).

### 4.6. Molecular Marker Analysis

Genomic DNA of *D. villosum* accession PI 491576, common wheat CS, CD-3, 22-12, and 24-20 was extracted from 2-week-old leaves by using the CTAB method [67]. V-genome-specific SCAR markers of DV1 were synthesized, as described by Zhang et al. [31]. The PLUG primers for the homologous group 3 and the EST primers specific to 3V#4 chromosomes were synthesized using the procedures described by Ishikawa et al. [43] and Li et al. [37], respectively. PCR was conducted using the T100^TM^ Thermal cycler (Bio-RAD Laboratories, Emeryville, CA, USA) in a 25 μL reaction mixture containing 2.5 μL of 10X buffer (50 mM KCl, 1.5 mM MgCl2, and 10 mM Tris-HCl; pH 8.3), 200 nmol of each dNTP, 100 ng of genomic DNA, 0.2 U of Taq polymerase (TianGen, Beijing, China), and 400 nmol of each primer. The cycling parameters were: 94 °C for 3 min for the initial denaturation, followed by 35 cycles of denaturation at 94 °C for 1 min, annealing at 55 °C (dependent on different primer sets) for 1 min, extension at 72 °C for 2 min, and a final extension at 72 °C for 10 min. The amplified products were separated on 2% (*w*/*v*) agarose gels and visualized by EtBr staining.

### 4.7. Assessment of Adult Plant Resistance to Stripe Rust

Thirty seeds from each line, including CD-3, 22-12, and 24-20 identified by ND-FISH, *D. villosum* accession PI 491576, and CS were sown in the field, and the plants were rated for stripe rust infection at the heading stage in the growing seasons during the years 2020 and 2021. The field test was conducted in Pixian city, Sichuan, China. CD-3 and *D. villosum* accession PI 491576 were used as resistance controls, and CS was used as the susceptible control. Ten seeds of each line were sown in a single-row plot. Three replicates were sown in an experimental block (3 m × 4 m), which was surrounded by a 30 cm-wide row of susceptible spreader. The mixed Pst strains, mainly CYR32, CYR33, and CYR34, that are currently prevailing in China [68], were used to infect the plants including the test lines and the spreader. Adult plant infection types (IT) (response of flag leaf) were recorded 18–20 days after inoculation based on a 0–4 scale, where 0, 0; 1, 2, 3, and 4 were considered to denote immune (I), near-immune (NI), high resistance (HR), moderate resistance (MR), moderate susceptibility (MS), and susceptibility (S), respectively [68]. The IT was recorded once every 10 days, totaling three times of recording. The *Pst* strains used were provided by the Plant Protection Institute, Sichuan Academy of Agricultural Sciences, China.

### 4.8. RNA-Seq and Transcriptome Assembly

Total RNA from CD-3 and *D. villosum* accession PI 491576 was extracted from the samples of 2-week-old leaves by using the TRIzol reagent (Invitrogen, Carlsbad, CA, USA). The RNA quality was determined using a NanoDrop 2000 spectrophotometer (Thermo Fisher Scientific, Wilmington, DE, USA). RNA-seq was performed by the Beijing Biomarker Technology Company, Beijing, China. High-quality clean reads were obtained from the raw reads after removing adaptor sequences, duplicated sequences, ambiguous reads (reads with “N” bases), and low-quality reads (reads with a length less than 20 bp). All reliable, clean reads were assembled into contigs by using the Trinity platform (http://trinityrnaseq.github.io, accessed on 10 April 2022) with the inchworm k-mer method [69]. The related contigs were clustered using TGICL software [70] to produce unigenes.

### 4.9. Development and Validation of 3V#3 Chromosome-Specific EST Markers 

The unigene fragments of CD-3 were compared with those of *D. villosum* accession PI 491576 to select the unigene sequences having high similarities (identity ≥ 90%) (designated as data pool 1). After removing the transcripts of CS from date pool 1 through reference genome matching, the candidate unigenes (data pool 2) for developing the 3V#3-specific markers were obtained. Subsequently, the candidate unigenes from data pool 2 were compared with CS sequences on www.ncbi.nlm.nih.gov, accessed on 10 April 2022. Primers were designed using Primer Premier 5.0 (PREMIER Biosoft, Palo Alto, CA, USA) based on the low-homologous region between candidate unigenes and those of CS, followed by synthesis by Shanghai Sangon Biotech Co., Ltd (Sangon Biotech, Shanghai, China). PCR was conducted in a T100TM Thermal cycler (Bio-RAD Laboratories) by using a 25 μL reaction system containing 2.5 μL of 10× buffer (50 mM KCl, 1.5 mM MgCl_2_, 10 mM Tris-HCl; pH 8.3), 40–100 ng of genomic DNA, 200 nmol of each primer, and 1 U of Taq DNA polymerase (TianGen Biotech, Beijing, China). The PCR protocol was as follows: initial denaturation at 94 °C for 5 min, followed by 35 cycles of denaturation at 94 °C for 1 min; annealing at 56 °C for 1 min (dependent on different primer sets); and extension at 72 °C for 2 min, followed by a final extension at 72 °C for 10 min. The amplicons were separated on a 1% (*w*/*v*) agarose gel and visualized by EtBr staining.

## 5. Conclusions

By using a combination of ND-FISH and PLUG markers, wheat–*D. villosum* 3V#3.3D translocation lines, 22-12 and 24-20, were identified. The line 22-12 exhibited strong stripe rust resistance, whereas the line 24-20 showed susceptibility to stripe rust, indicating that the *YrCD-3* is located on the short arm of 3V#3S. Furthermore, the line 22-12 could be a valuable resource for both basic and applied research for wheat resistance breeding. The 3V#3S-specific EST markers developed in the current study are available for marker-assisted selection in wheat breeding. 

## Figures and Tables

**Table 1:** PLUG primers belonging to *Triticeae* homoeologous Group 3 used in the study.

Marker Name	Primer Sequence (5′–3′)	Wheat Bin Map Location	Wheat Chromosomal Location	Restriction Enzymes	Length of 3V Bands, bp
TNAC1248	F: ATGATGCAGCAGCAAATTACA	3AS4-0.45-1.00	3AS-211.50	TaqI	1000
	R: CTGAGGAGCCTCTCCAACTCT	C-3BS1-0.33	3BS-251.65		
		3DS3-0.24-0.31	3DS-172.90		
TNAC1326	F:ACAGATCGAGATGTTTATTGAAA	3AS4-0.45-1.00	3AS-137.91	TaqI	750
	R: GATCAAAGAGATGCGCTGAAG	3BS1-0.33-0.57	3BS-181.49		
		3DS10-0.31-0.44	3DS-127.34		
TNAC1267	F: GAGAGGCAGCTTCACTAGCAG	3AL3-0.42-0.61	3AL-522.17	TaqI	200
	R: CGTCAGGATCAGCTCTCATGT	3BL2-0.22-0.41	3BL-527.29		
		3DL1-0.23-0.81	3DL-401.87		
TNAC1359	F: GTAAATAGCGCCATCTGCGTA	3AL3-0.42-0.61	3AL-531.08	TaqI	750
	R: CTCTGGATGCAGTTGGAATGT	3BL3-0.41-0.50	3BL-547.12		
		3DL1-0.23-0.81	3DL-419.90		

**Table 2:** Localization of the new markers and *D. villosum#*4-specific markers in *D. villosum#3*, their primer sequences and product sizes.

Primers No.	Primer Sequences(5’–3’)		Localization in *D. villosum*#3	Localization in Wheat	Product Size in *D. villosum*#3 (bp)	Product Size in *D. villosum*#4 (bp)
	Forward	Reverse				
3V#3-1	GCCTCATGCGGCTGTTGG	GAGGTCATGGTGAGCACGAGA	3V#3S	3BS	148	-
3V#3-2	CGGCAAGAGGTCGATGGT	TGACCCACGCACGCACTA	3V#3L	1DS	210	-
3V#3-3	GCGTCTTGGATGTCCTG	CGATTTGCTGCCCTACAT	3V#3L	3AL	177	-
3V#3-4	GCATGATAGAGAGGTTAGCCAT	CACTGGTGATGTTGTTCAGTACT	3V#3S	1AL	90	-
3V#3-5	TCGATACAATTGTTCTTGAGATATG	ACTGGTGCCCTCTTGACG	3V#3L	3AL	560	-
3V#3-6	AAACAATCTAGCACTACCCAGAGG	AAGAGGAAGAGAAATAAGCGAGG	3V#3L	3DL	166	-
3V#3-7	AGGTCCTTGTCCGAGGTGAT	ATGTTACCGATACTGATGCCACT	3V#3S	3AS	982	-
3V#3-8	ATGCTGAACGCAAGGTCAAATA	TGCTGAAGCCCATCACGAAG	3V#3L	3DL	1500	-
3V#3-9	CGACTGGTCCACCGTTTC	CGCTGCCTAGTTACCTCTGTT	3V#3L	3DL	1700	-
3V#3-10	CTTTCAAGGTAATCCCAGAACT	TGGAAAGCAAACAGGATACG	3V#3S	3AS	126	-
3V#3-11	TACGAATAACAACTGCAAGCAGAAT	TGCTAATGGCATCAGCGTCA	3V#3L	3DL	1302	-
3V#3-12	CGACTGGTCCACCGTTTC	CGCTGCCTAGTTACCTCTGTT	3V#3L	3AL	1700	-
3V#4-1 *	TCCATCATAGCACCTTCAGACTCAAG	GACAACTCGGCAATCACCAAGGA	3V#3S	5BL	**850**	**377**
3V#4-2 *	GGCAACTCAAATTATAGGATCACGAC	GCAAGGCGGAGTAGCTCACA	3V#3L	5DL	243	243
3V#4-3 *	TTCGTCATCTTTGTTGACATGGCAA	GCAAGGCGGAGTAGCTCACA	3V#3L	5DL	**240**	**263**
3V#4-5 *	TTAGAGCGACGACAACTATGC	CAGTATAAGATAGGAGAAGCGACAG	3V#3L	3AL	175	175
3V#4-7 *	GTTATATCGGTTGAGGCGTCTATAC	AACAGTGAGTTCTTCAGGACAGA	3V#3L	3DL	**700**	**341**
3V#4-11 *	CTCGTCGGTCTCAGAAGTCAA	TCCACAGAATCATCGGCTCTC	3V#3L	3DL	760	760
3V#4-12 *	CCTCCTCTTCCTCCTCTTCC	TCGCACCATCACCGTACTT	3V#3L	3DL	499	499
3V#4-14 *	GGACGGATGTAGTCTTGTTCAA	CTCGTATCGTACTGCTACTCA	3V#3L	3DL	593	593
3V#4-16 *	GTCCACCAAATCACATCAAACA	GCTCTCACAAGTCACAACAATT	3V#3L	3DL	180	180
3V#4-17 *	ACCATATACTTCGGTGGAACATAC	GCATAGTTACTCTATCACAGACTCA	3V#3L	3DL	304	304
3V#4-18 *	TACCATATACTTCGGTGGAACATAC	GCATAGTTACTCTATCACAGACTCA	3V#3L	3DL	305	305
3V#4-19 *	TGCTCTTCACAGTTCATCTCCT	AGACAAGTTCAGTTCCACACTC	3V#3L	3DL	364	364
3V#4-20 *	TGGTTGCTTCTCAGTTGTGTTG	TACTCGGATAGTGCCTTGTTGA	3V#3S	3AS	**600**	**237**
3V#4-21 *	GTTGCTTCTCAGTTGTGTTGGA	TACTCGGATAGTGCCTTGTTGA	3V#3S	3AS	**600**	**235**
3V#4-22 *	TGGTTGCTTCTCAGTTGTGTTG	CGGATAGTGCCTTGTTGATGAC	3V#3S	3AS	**600**	**233**
3V#4-24 *	GAGAACTGCTCAACATGACAATAAG	CAACAGTATCATCAATGGAGGTCTT	3V#3S	3DS	144	144
3V#4-26 *	TCGCCAGCACCAACCAAT	CAGCACAGCACACCAATGAA	3V#3S	3DS	686	686
3V#4-27 *	GTGACACCAATAGAAGGCAGAA	GGAGGAGCATACCGTGGAA	3V#3S	3AS	403	403

* indicates primers reported by Li et al. [37]. Bold font means different products’ sizes between *D.villsoum*#3 and *D. villosum*#4.

## Data Availability

The data presented in this study are available on request from the corresponding author.

## Supplementary Materials

The following supporting information can be downloaded at: https://www.mdpi.com/article/10.3390/plants11101329/s1, Figure S1: Irradiation-induced chromosomal aberrations and survival rate in M_0_. (A–D): Feulgen staining of CD-3^200Gy^ (A) and CD-3^ck^ (B–D); (E): Survival rate of CD-3^200Gy^ and CD-3^ck^. The black arrows indicate the chromosome segments, and the red arrows indicate the chromatin bridge.; Figure S2: PCR products of DV1 and summary of chromosomal aberrations in M_1_. (A), PCR amplification generated by the marker DV1; (B), FISH karyotype of aberrant chromosomes in M_1_. M, Trans2k plus DNA markers (Trangene, Beijing, China); 1, common wheat CS “Chinese Spring”; 2, CD-3; 3, *D. villosum*; 4–24, partial M_0_ plants; the arrow indicates *D. villosum*-specific bands. Type 1 indicates the 3V#3.wheat translocations, type 2 indicates the 3V#3.3V#3 translocation, and type 3 indicates the 3V#3 deletions. All chromosomes in (B) were stained with DAPI (blue), Oligo-pTa535 (red), Oligo-pSc119.2 (green) and Oligo-pHv62-1(white).
