# Supplementary figures and images for: Molecular Cytogenetic Identification of the Wheat–Dasypyrum villosum T3DL·3V#3S Translocation Line with Resistance against Stripe Rust
Source: Plants (Basel). 2022 May 18;11(10):1329. doi: 10.3390/plants11101329 (PMC9145344; doi:10.3390/plants11101329)

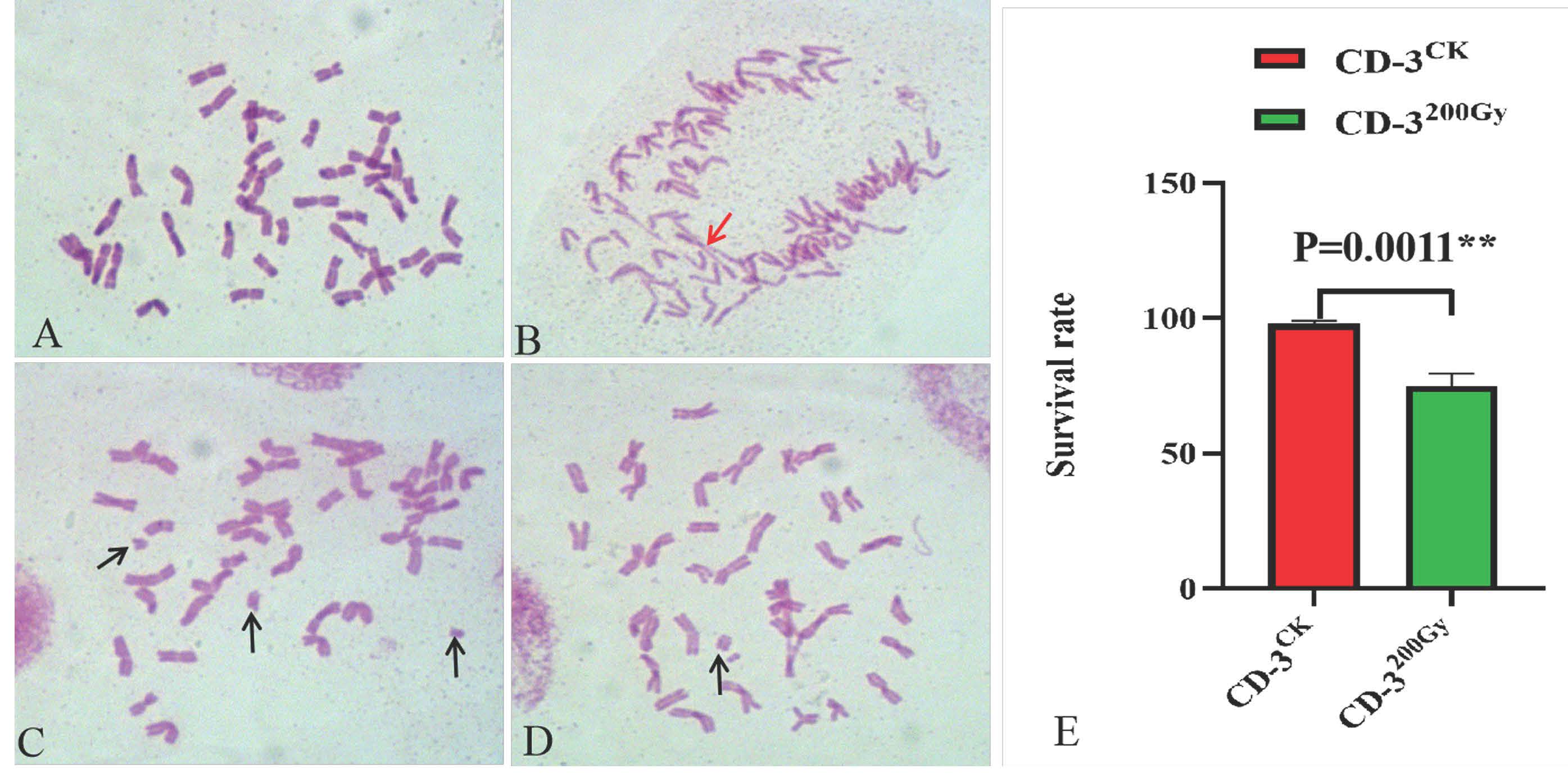

Supplement: Supplementary file 1 [file plants-11-01329-s001.zip › Figure S1.jpeg]

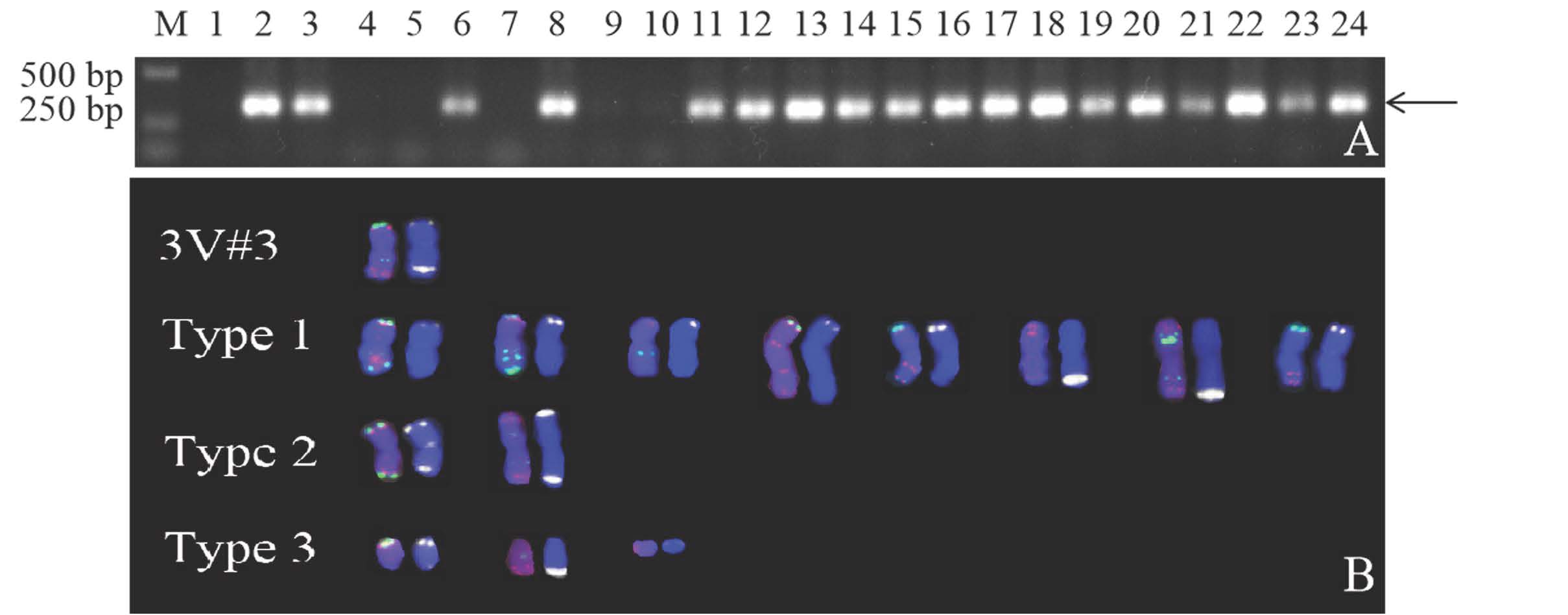

Supplement: Supplementary file 1 [file plants-11-01329-s001.zip › Figure S2.jpeg]
